# Elevated CXCL12 expression in the bone marrow of NOD mice is associated with altered T cell and stem cell trafficking and diabetes development

**DOI:** 10.1186/1471-2172-9-51

**Published:** 2008-09-15

**Authors:** Qibin Leng, Yuchun Nie, Yongrui Zou, Jianzhu Chen

**Affiliations:** 1 Koch Institute for Integrative Cancer Research and Department of Biology, Massachusetts Institute of Technology, Cambridge, MA 02139, USA; 2 Department of Microbiology, Columbia University, New York, NY 10032, USA; 3 Institut Pasteur of Shanghai, Chinese Academy of Science, Shanghai, 200025, PR China

## Abstract

**Background:**

Type I diabetes (TID) is an autoimmune disease resulting from destruction of the insulin-producing β-cells by autoreactive T cells. Studies have shown that polymorphisms of chemokine CXCL12 gene are linked to TID in humans. In non-obese diabetic (NOD) mice, which are predisposed to develop the disease, reduction of CXCL12 level leads to significant delays in the onset of diabetes. Despite these initial observations, however, how CXCL12 affects development of TID has not been fully investigated.

**Results:**

We found that the level of CXCL12 transcript is significantly elevated in the bone marrow of NOD mice as compared to Balb/c and C57BL/6 mice. Correspondingly, naïve T cells, regulatory T cells and hematopoietic stem cells (HSC) accumulate in the bone marrow of NOD mice. Treatment of NOD mice with AMD3100, an antagonist for CXCL12's receptor CXCR4, mobilizes T cells and HSC from the bone marrow to the periphery, concomitantly inhibits insulitis and delays the onset of diabetes.

**Conclusion:**

These results suggest that the elevated CXCL12 expression promotes TID in NOD mice by altering T cell and hematopoietic stem cell trafficking. The findings highlight the potential usefulness of AMD3100 to treat or prevent TID in humans.

## Background

Type I diabetes (TID) is an autoimmune disease, resulting from destruction of the insulin-producing β-cells in the islets of Langerhans by autoreactive T cells. The non-obese diabetic (NOD) mice, which are predisposed to develop the disease, have served as a model for studying the mechanism, pathogenesis and interventions of the human disease [1,2]. In NOD mice, a unique major histocompatibility complex (MHC) class II allele (IA^g7^) in combination with a defect in the programmed cell death pathway is thought to permit autoreactive T cells to escape negative selection in the thymus [3-5]. In the periphery, additional defects in peripheral tolerance mechanisms enable activation of the autoreactive T cells [6-8], leading to the disease development.

Besides development and activation of autoreactive T cells, factors that regulate T cell trafficking likely contribute to the disease development because destruction of β-cells requires infiltration of autoreactive T cells into the islets. Chemokines are a group of low molecular weight proteins and regulate cell trafficking by binding to specific G-protein-coupled seven-span transmembrane receptors on target cells. For example, CXCL12, also known as stromal cell derived factor-1 (SDF-1), and its receptor CXCR4 play a critical role in regulating hematopoietic cell trafficking [9]. It is required for fetal liver-derived hematopoietic stem cells (HSC) to colonize the bone marrow during embrogenesis and retention/homing of these cells in the bone marrow in the adult. It also regulates trafficking of many other cell types that express CXCR4, including lymphocytes and cancer stem cells. In humans, polymorphisms in CXCL12 gene are linked to TID [10-12]. In NOD mice, the onset of diabetes is significantly delayed by reducing the level of CXCL12 either by antibody-mediated neutralization or G-CSF-induced suppression of CXCL12 transcription [13-16]. Despite these initial observations, however, how chemokine CXCL12 affects development of TID has not been fully investigated.

In this report, we show that expression of chemokine CXCL12 is elevated in the bone marrow of NOD mice, resulting in accumulation of both T cells and HSC in the bone marrow. Treatment of NOD mice with CXCR4 antagonist AMD3100 [17] mobilizes T cells from the bone marrow to peripheral lymphoid tissues, and significantly delays the onset of insulitis and diabetes. Our findings suggest that the elevated CXCL12 expression in the bone marrow likely promotes TID in NOD mice by altering T cell trafficking and stem cell mobilization.

## Results

### Naive T cells accumulate in the bone marrow of NOD mice

**Figure 1:**
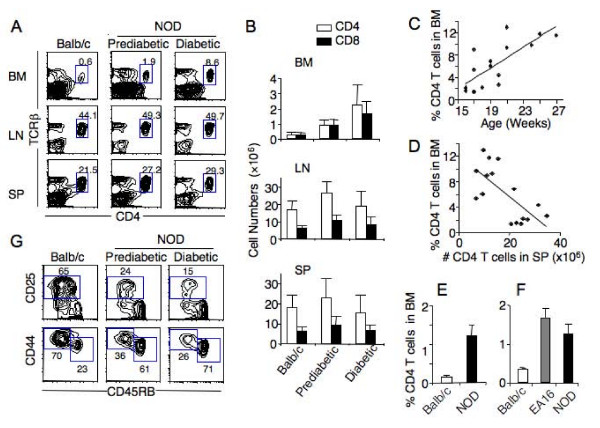
**Accumulation of naïve T cells in the bone marrow of NOD mice**. A. Frequency of CD4 T cells in the bone marrow of Balb/c, prediabetic and diabetic NOD mice. Cells from bone marrow (BM), spleen (SP) and pooled lymph nodes (LN, including cervical, mediastinal, auxiliary, brachial, mesenteric, and inguinal nodes) were stained for CD4, CD8, TCRβ, and PI. CD4 versus TCRβ profiles are shown for live cells. The numbers indicate the percentages of CD4^+^TCR^+ ^cells in the gated regions. p < 0.001 comparing percentages of CD4 T cells in the bone marrow between Balb/c and NOD mice. B. The average number of CD4 (open bar) and CD8 (solid bar) T cells in the BM, LN and SP of Balb/c (n = 22), prediabetic (n = 19) and diabetic (n = 16) NOD mice. p < 0.01 comparing CD4 or CD8 T cells in the bone marrow between Balb/c and prediabetic or diabetic NOD mice. C. Increase of percentages of CD4 T cells with age in the bone marrow of diabetic NOD mice. The r^2 ^value is 0.76. D. Inverse correlation between percentage of CD4 T cells in the bone marrow and CD4 T cell number in the spleen of the same diabetic NOD mice. The r^2 ^value is -0.69. Data presented in C and D are from the same group of mice. One dot represents one mouse. E. Comparison of percentages of CD4 T cells in the bone marrow of 4–5 week old BLAB/c (n = 6) and NOD (n = 8) mice. p < 0.001. F. Comparison of percentages of CD4 T cells in the bone marrow of 6–9 week old Balb/c (n = 5), EA16 (n = 3) and NOD (n = 8) mice. p < 0.001 comparing Balb/c to NOD (or EA16) mice. G. Phenotype of bone marrow T cells. Bone marrow cells were stained for TCR, CD4, CD25 plus CD45RB, or for TCR, CD4, CD44 and CD45RB. Representative plots from at least four independent experiments are shown, gating on CD4^+^TCR^+ ^cells.

Compared to age-matched Balb/c or C57BL/6 mice, prediabetic NOD mice (15–16 week-old and no detectable urine glucose) had a significantly higher percentage of CD4^+ ^T cells (~3-fold) in the bone marrow (Figure 1A and data not shown). The increase was even more pronounced in diabetic NOD mice (23 week-old, urine glucose ≥ 500 mg/dl), reaching up to 15-fold of that in Balb/c mice (Figure 1A). Correspondingly, the number of CD4 T cells in the bone marrow of prediabetic NOD mice (0.86 ± 0.38 × 10^6^) was 3 times higher than that in the Balb/c bone marrow (0.29 ± 0.16 × 10^6^), although both bone marrows had similar number of cells (41.6 ± 11.1 × 10^6 ^versus 46.4 ± 13.3 × 10^6^, p < 0.09) (Figure 1B). In diabetic NOD mice, the percentages of CD4 T cells in the bone marrow increased with age (Figure 1C) and the increase was correlated with a decrease of CD4 T cell number in the spleen (Figure 1D). A similar increase in the percentage and number of CD8 T cells was also observed in the bone marrow of NOD mice (Figure 1B and data not shown).

To investigate the role of inflammation or hyperglycemia in the T cell accumulation in the bone marrow of NOD mice, we used 4–5 week-old NOD mice, in which insulitis (inflammation) was minimal. As shown in Figure 1E, the percentage of CD4 T cells in the bone marrow was still significantly higher (1.2 ± 0.3%) than in age-matched Balb/c mice (0.2 ± 0.03%). Furthermore, in EA16 mice, which do not develop diabetes because the transgenic expression of an I-E molecule confers almost complete protection from insulitis of NOD mice [18], the percentage of CD4 T cells in the bone marrow was significantly increased as compared to Balb/c mice (Figure 1F). In addition, most CD4 T cells in the bone marrow of prediabetic NOD mice were CD44^lo^CD45RB^hi^CD25^- ^naïve T cells (Figure 1G). The proportion of the naïve T cells increased as the NOD mice became diabetic. Together, these results show that naïve T cells accumulate in the bone marrow of NOD mice.

### Increased homing leads to T cell accumulation in the bone marrow of NOD mice

**Figure 2:**
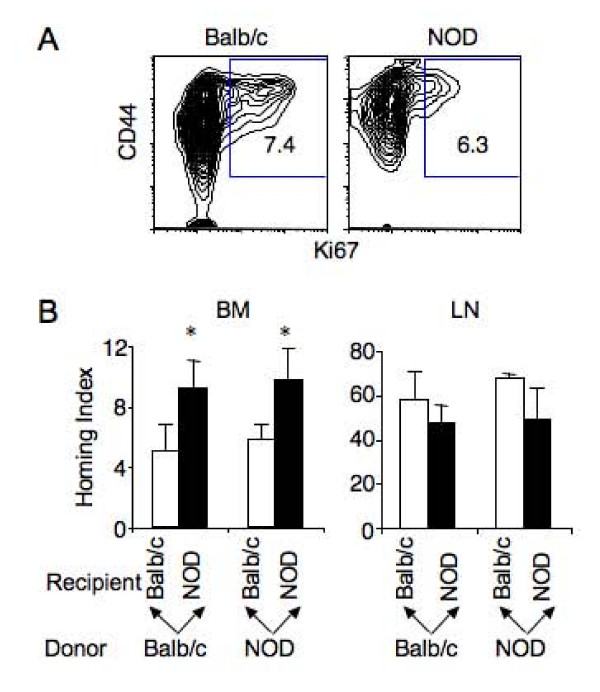
**T cell accumulation in the bone marrow of NOD mice is due to homing**. A. Comparison of steady-state level of CD4 T cell proliferation in the bone marrow of 15–16 week-old Balb/c and prediabetic NOD mice. Bone marrow cells were stained for TCR, CD4, CD44 and Ki67. Representative Ki67 versus CD44 expression profiles are shown for TCR^+^CD4^+ ^cells. The numbers are percentage of Ki67^+ ^cells. B. Comparison of T cell homing to the bone marrow and lymph nodes in Balb/c and NOD mice. CFSE-labeled T cells from Balb/c or NOD mice were injected intravenously into both Balb/c and NOD recipients (12–16 week-old) and analyzed 2 hours following the transfer. Homing index is calculated by dividing percentage of CFSE-positive donor CD4 T cells in the bone marrow or lymph nodes by that in the spleen of the same recipient. Mean ± SD of homing index of CD4 T cells in at least four mice per group is shown. *p < 0.01.

To determine if T cell accumulation in the bone marrow of NOD mice was due to proliferation, T cells in the bone marrow were stained for cycling marker Ki67. No significant difference in the percentages of Ki67^+^proliferating CD4 T cells was detected in the bone marrow of prediabetic NOD mice and age-matched Balb/c mice (Figure 2A). As in Balb/c mice, the proliferating CD4 T cells in the bone marrow of NOD mice were CD44^hi ^effector or memory T cells.

To investigate the role of recruitment in T cell accumulation in the bone marrow, total T cells were purified from NOD and Balb/c mice, labeled with CFSE, and transferred into both NOD and Balb/c mice. Distribution of CFSE-positive donor CD4 T cells in the recipients was assayed 2 hours following the transfer. No significant difference was detected in T cell distribution in the lymph nodes between Balb/c and NOD mice when either NOD or Balb/c T cells were transferred (Figure 2B right panel), demonstrating that T cells from Balb/c and NOD mice have equivalent motility. However, significantly more transferred Balb/c and NOD T cells were detected in the bone marrow of NOD than Balb/c mice (Figure 2B left panel). Forty-eight hours after the transfer, the difference between T cell distribution in the bone marrow of NOD and Balb/c recipients was even greater (see Additional file 1). Therefore, enhanced recruitment likely contributes to the accumulation of naïve T cells in the bone marrow of NOD mice. Because the enhanced recruitment is restricted to the bone marrow and independent of the source of the donor T cells, factors that mediate the preferential homing of naïve T cells likely reside in the bone marrow of NOD mice.

### Elevated CXCL12 expression promotes T cell recruitment to the bone marrow of NOD mice

**Figure 3:**
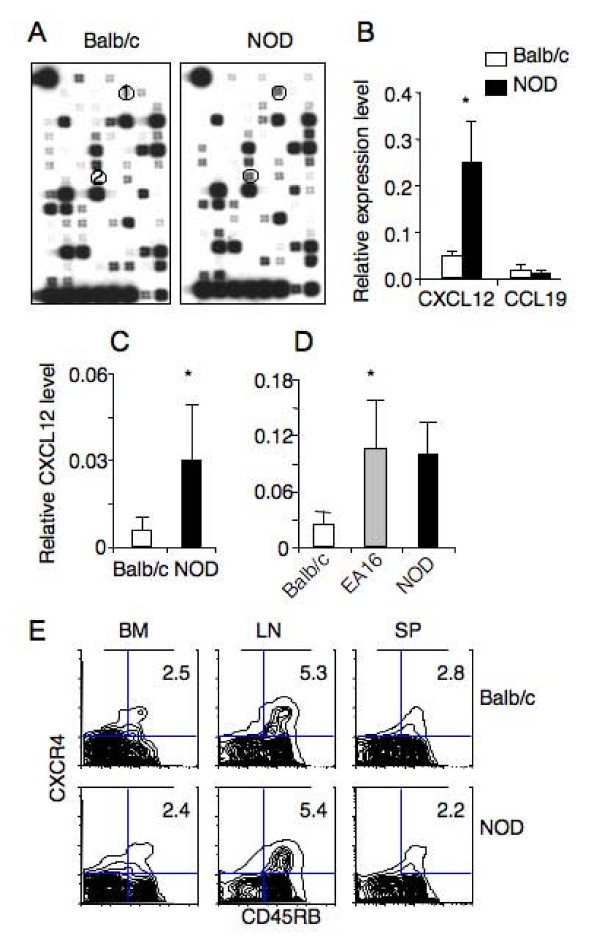
**Elevated CXCL12 expression correlates with T cell accumulation in the bone marrow of NOD mice**. A. Analysis of chemokine expression in the bone marrow. RNA was isolated from bone marrow of Balb/c (n = 5) and prediabetic NOD (n = 6) mice, pooled, labeled and used as a probe to hybridize with GEArray chemokine array filters. Representative chemiluminescent images from the hybridization are shown. 1, CCL19; 2, CXCL12. B. Quantitation of CXCL12 and CCL19 transcripts by RT-PCR. The same RNA as in A was used in real-time RT-PCR analysis for CXCL12, CCL19 and GAPDH. The relative transcript levels of CXCL12 and CCL19 to GAPDH are shown. C. Comparison of CXCL12 transcript levels in the bone marrow between 4–5 week-old NOD mice (n = 8) and Balb/c mice (n = 6). D. Comparison of CXCL12 transcript levels in the bone marrow among 6–9 week-old Balb/c (n = 5), EA16 (n = 3) and NOD (n = 8) mice. E. CXCR4 expression by CD4 T cells in different organs of Balb/c and prediabetic NOD mice. Cells from BM, LN and SP of Balb/c and prediabetic NOD mice were stained for TCR, CD4, CD45RB and CXCR4. Representative CXCR4 versus CD45RB profiles are shown for TCR^+^CD4^+ ^cells. The numbers indicate percentages of cells in the gated areas. * p < 0.05.

To identify chemokines that mediate the observed preferential T cell homing, we compared chemokine transcription in the bone marrow between NOD and Balb/c mice using Mouse Chemokines & Receptors Microarrays. Only chemokine CXCL12 and CCL19 transcripts were elevated in the bone marrow of NOD mice as compared to Balb/c mice (Figure 3A). Quantitative RT-PCR analysis showed that the CXCL12 transcript was 3–5 fold higher in the bone marrow of NOD than Balb/c mice (Figure 3B), whereas the difference in the level of CCL19 transcript was not confirmed. Consistently, the level of CXCL12 transcript was significantly higher in the bone marrow of 4–5 week-old NOD mice and EA16 mice than their respective age-matched controls (Figure 3C and 3D). In addition, majority of CD4 T cells in the spleen, lymph nodes, and bone marrow of NOD mice that expressed CXCR4 were CD45RB^+ ^naïve T cells (Figure 3E), consistent with their accumulation in the bone marrow. Although only a few percentages of CD4 T cells stained positive for surface CXCR4, all CD4 T cells from both NOD and C57BL/6 mice were positive for intracellular staining of CXCR4 (data not shown).

**Figure 4:**
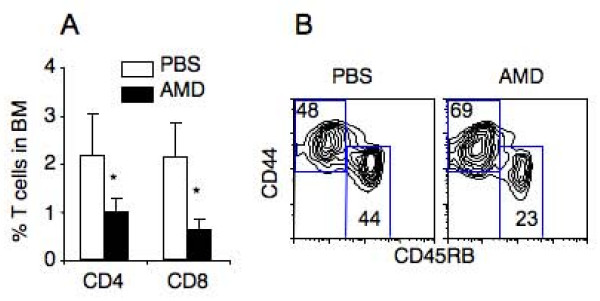
**AMD3100 inhibits naive T cell accumulation in the bone marrow of NOD mice**. Prediabetic NOD mice (15–16 weeks of age) were treated with PBS or AMD3100 (AMD) daily for 8 days. Two hr after the last AMD3100 injection, mice were analyzed by flow cytometry. A, Comparison of percentage of CD4 and CD8 T cells (mean ± SD) in the bone marrow of AMD3100 (n = 4) and PBS (n = 6) treated NOD mice. * p < 0.05. B, Comparison of CD44 versus CD45RB profiles of TCR^+^CD4^+ ^cells from AMD3100 (n = 4) and PBS (n = 6) treated NOD mice. The numbers indicate percentages of cells in the gated areas. The percentages (mean ± SE) of CD45RB^hi ^CD44^lo ^naïve T cells are 47.3 ± 10.9 for PBS-treated mice and 24.8 ± 3.2 for AMD3100-treated mice (p < 0.01).

AMD3100 is a low molecular weight antagonist of CXCR4 [17]. If interaction of CXCL12 with CXCR4 is critical for the observed T cell accumulation, treatment of NOD mice with AMD3100 should inhibit T cell accumulation in the bone marrow. Thus, prediabetic NOD mice were given AMD3100 (5 mg/kg) daily for 8 days and the distribution and phenotype of T cells in their bone marrow were assayed. AMD3100 treatment significantly reduced the proportion of both CD4 and CD8 T cells in the bone marrow of the prediabetic NOD mice as compared to prediabetic NOD mice that were given PBS (Figure 4A). In particular, the proportion of CD4 T cells with the naïve phenotype (CD45RB^hi ^CD44^lo^) was preferentially reduced in the AMD3100 treated NOD mice (Figure 4B). Together with CXCL12's known function in regulating T cell migration [19], these results demonstrate that the elevated CXCL12 expression results in the accumulation of naïve T cells in the bone marrow of NOD mice.

### Elevated CXCL12 expression promotes recruitment/retention of regulatory T cells (Tregs) and hematopoietic stem cells in the bone marrow

**Figure 5:**
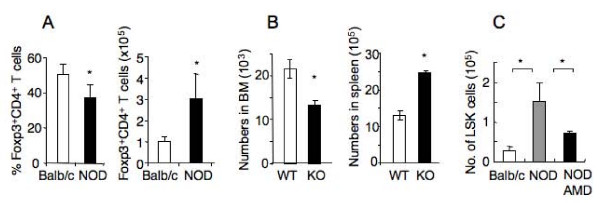
**The elevated CXCL12 expression promotes recruitment/retention of Treg and hematopoietic stem cells in the bone marrow**. A. Comparison of the percentages and numbers of Foxp3^+^CD4^+ ^Tregs in the bone marrow between age-matched Balb/c (n = 7) and prediabetic NOD (n = 20) mice (15 weeks of age). Bone marrow cells were assayed for TCR, CD4 and Foxp3. Tregs are identified as TCR^+^Foxp3^+^CD4^+ ^cells. The percentage value of Foxp3^+ ^cells is expressed as percent of CD4^+ ^T cells. B. Effect of CXCR4 deletion on Treg distribution in the spleen and bone marrow. Cells from spleen and bone marrow of *Cxcr4*^*f/f *^Lck-Cre (KO) mice and littermate *Cxcr4*^+/*f *^Lck-Cre (WT) (8 weeks of age) were assayed for TCR, CD4, CD25 and Foxp3. The numbers of TCR^+^CD4^+^CD25^+^Foxp3^+ ^cells are compared in the spleen and bone marrow from 3 mice per group. C. Comparison of numbers of hematopoietic stem cells (Lin^-^Sca1^+^c-Kit^+^, LSK) among age-matched Balb/c (n = 5), prediabetic NOD (n = 5) and AMD3100-treated (8 days) prediabetic NOD mice (n = 4). *p < 0.05.

The elevated CXCL12 expression is expected to promote recruitment or retention of other cell types/subsets that express CXCR4 in the bone marrow of NOD mice. Although no significant difference in percentage or number of NKT cells, dendritic cells or B cells was detected in the bone marrow between NOD and Balb/c mice (data not shown) nor any change in distribution of these cell types in NOD mice following AMD3100 treatment (see Additional file 2 and data not shown), the numbers of Foxp3^+^CD4^+ ^Tregs were consistently higher in the bone marrow of prediabetic NOD mice than Balb/c mice (Figure 5A). A lower percentage of CD4 T cells that are Tregs in the bone marrow of NOD mice as compared to Balb/c mice likely reflects the greater accumulation of naïve CD4 T cells than Tregs in the NOD bone marrow. To unequivocally determine the involvement of CXCL12-CXCR4 interaction in regulating Treg trafficking, we used mice in which CXCR4 expression was specifically inactivated in T cells via Cre-mediated recombination [20]. In the absence of CXCR4, the numbers of Treg in the bone marrow was decreased significantly whereas the number of Treg in the spleen was increased as compared to littermate wildtype mice (Figure 5B). Thus, Treg trafficking into the bone marrow is partly regulated by CXCL12.

CXCL12 plays a critical role in retention of HSC in the bone marrow of adult mice [9,21]. As expected, the number of Lin^-^Sca1^+^c-Kit^+ ^HSC was significantly higher in the bone marrow of prediabetic NOD mice than in age-matched Balb/c mice (Figure 5C). Following AMD3100 treatment for 8 days, the number of HSC in the bone marrow of NOD mice was significantly reduced.

### Interference of CXCR4 function delays the development of diabetes in NOD mice

**Figure 6:**
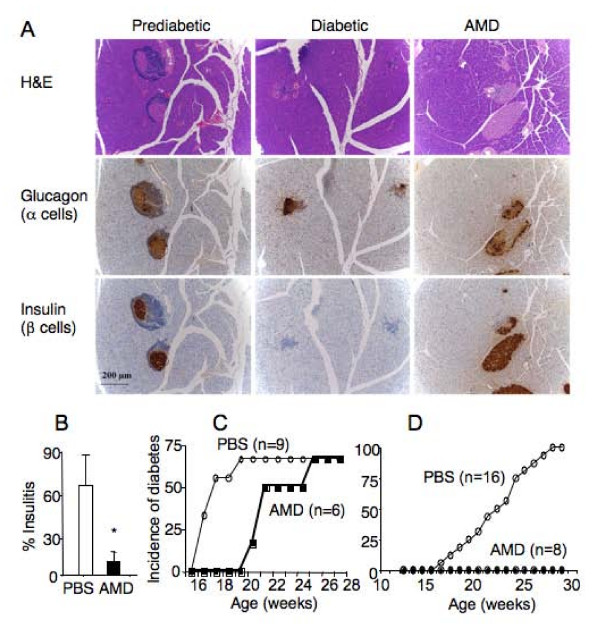
**AMD3100 treatment inhibits leukocyte infiltration and development of diabetes in NOD mice**. A. Immunohistological staining of pancreatic sections of NOD mice. The pancreas of prediabetic, diabetic NOD mice or prediabetic NOD mice that have been given AMD3100 for 8 days were fixed and embedded. Parallel tissue sections were stained with haematoxylin and eosin (H&E, top panel), anti-glucagon (middle panel) or anti-insulin (bottom panel) antibodies. Note lymphocyte infiltration in the islets of prediabetic NOD mouse without AMD3100 treatment. B. Percentage of insulitis in pancreatic sections of 15 week-old prediabetic NOD mice that were given daily with PBS (n = 8) or AMD3100 (n = 6) for 8 days. *p < 0.01. C and D. Comparison of diabetes incidence in NOD mice that were given AMD3100 or PBS for 3 weeks (C) or 14 weeks (D), starting at 15–16 weeks of age. Number of mice in each group (n) is shown. Mice are scored as diabetic when glucose level in the urine reaches 500 mg/dl. p < 0.01.

To determine the relationship between CXCL12-mediated dysregulation of T cell and stem cell trafficking and development of diabetes, we treated NOD mice with AMD3100 and monitored progression of the disease. As shown in Figure 6A, prediabetic NOD mice had significantly more insulin-expressing islets than diabetic NOD mice (26.7 ± 5.2 per section versus 15.8 ± 9.4 per section, p < 0.05). Among age-matched prediabetic NOD mice, AMD3100 treatment (daily for 8 days) significantly reduced lymphocyte infiltration into the islets (insulitis) (Figure 6A and 6B). When prediabetic NOD mice (15–16 weeks of age) were given AMD3100 or PBS daily for three weeks, PBS-treated NOD mice rapidly developed diabetes between 17 and 20 weeks of age as indicated by glucose levels in the urine (>500 mg/dl) (Figure 6C). In contrast, none of the AMD3100-treated mice developed glycosuria disease during the same period. However, two weeks after AMD3100 treatment was terminated, AMD3100-treated mice began to develop diabetes and the incidence reached the same level (70%) as PBS-treated mice by 26 weeks of age. To test whether continuous AMD3100 treatment can inhibit the development of diabetes, prediabetic NOD mice (15 weeks of age) were given AMD3100 daily for 14 weeks. PBS-treated NOD mice started to show evidence of diabetes at 16 weeks of age and by 29 weeks of age, all mice developed diabetes (Figure 6D). In contrast, none of the AMD3100-treated mice developed the disease during the same period. These results show that AMD3100 treatment inhibits leukocyte infiltration in the islets (insulitis) and continuous treatment delays development of overt diabetes.

## Discussion

We show here that among all chemokines examined the level of CXCL12 transcript, which is tightly correlated with the protein level [14], is significantly elevated in the bone marrow of NOD mice as compared to Balb/c mice. CXCL12 specifically stimulates chemotaxis of cells that express CXCR4 [20,22,23]. The elevated CXCL12 levels are therefore expected to have a significant effect on recruitment and retention of CXCR4-expressing cells in the bone marrow of NOD mice. Based on analyses of young, prediabetic, diabetic NOD mice and EA16 mice, the phenotype of CXCR4-expressing T cells, the effect of AMD3100 treatment, and direct recruitment assay, our studies unequivocally demonstrate that the elevated CXCL12 expression results in recruitment and accumulation of naïve T cells in the bone marrow of NOD mice. Because there is no difference in the proportion of T cells that express CXCR4 between NOD and Balb/c mice, and because Balb/c T cells are also preferentially recruited to the NOD bone marrow, the observed recruitment and accumulation of naïve T cells in the bone marrow of NOD mice appears to be due solely to the elevated CXCL12 expression. In addition, our results show that the elevated CXCL12 expression also leads to the accumulation/retention of Tregs and HSC in the bone marrow.

What is the relationship between the elevated CXCL12 expression in the bone marrow and development of diabetes in NOD mice? The elevated CXCL12 expression is unlikely a consequence of inflammation or hyperglycemia associated with insulitis or diabetes in NOD mice because CXCL12 transcript was detected in the bone marrow of EA16 mice and young (4–5 week old) NOD mice. More likely, NOD mice are predisposed to express elevated CXCL12 in the bone marrow. Conversely, because EA16 mice do not develop diabetes despite the elevated CXCL12 expression and accumulation of T cells in the bone marrow, the elevated CXCL12 expression alone is insufficient to initiate diabetes in the absence of autoreactive T cells. Nevertheless, the elevated CXCL12 expression likely promotes diabetes development. In humans, polymorphisms in CXCL12 gene are linked to susceptibility to TID [10-12]. Using allele-specific transcript quantification in Epstein-Barr virus-transformed lymphoblastoid cell lines, one study reported evidence that polymorphisms have a cis-acting effect on CXCL12 transcription [24]. However, whether TID patients with specific CXCL12 polymorphisms have elevated CXCL12 expression has not been reported. In NOD mice, neutralization of CXCL12 by administration of antibody suppresses insulitis and delays the onset of diabetes [15]. Similarly, administration of G-CSF, a cytokine known to induce suppression of CXCL12 transcription, also reduces insulitis and diabetes in NOD mice [15,16]. Consistent with these previous observations, we found that the elevated CXCL12 expression and its consequent effect on T cell trafficking are positively correlated with the disease progression in NOD mice. Complete Freund adjuvant (CFA), which is known to inhibit diabetes development [25,26], also inhibits CXCL12 expression [27] and T cell accumulation in the bone marrow of NOD mice (see Additional file 3). Finally, treatment of prediabetic NOD mice with AMD3100 abolishes T cell accumulation in the bone marrow and simultaneously inhibits disease development. Together, these findings strongly suggest that CXCL12 promotes development of diabetes in NOD mice and perhaps in humans.

Our findings also suggest potential mechanisms by which the elevated CXCL12 expression promotes disease progression in NOD mice. We found that the elevated CXCL12 expression leads to homing of naïve T cells to the bone marrow. A recent study showed that diabetogenic T cells also preferentially home to the bone marrow of NOD mice [28], perhaps as a result of the elevated CXCL12 expression shown here. It is possible that accumulation of T cells in the bone marrow of NOD mice might lead to lymphopenia in the peripheral lymphoid organs and homeostatic proliferation and differentiation of autoreactive T cells [29-32], which in turn promotes diabetes development. It is also possible that elevated CXCL12 expression may promote disease progression in NOD mice through its effect on trafficking of autoreactive T cells, especially into islets. Studies have shown that CXCL12-CXCR4 interaction is required for recruitment of autoreactive T cells to rheumatoid arthritis synovium and the inflamed joint of collagen-induced arthritis [33,34]. In a virus-induced TID model, blockade of CXCL10 (IP-10) prevents diabetes development by impeding expansion of autoreactive T cells and their migration into the pancreas [35]. We showed that inhibition of CXCR4 by AMD3100 significantly reduced insulitis (Figure 5B). Although we did not detect any difference in CXCL12 expression in pancreas between prediabetic NOD mice and BALB/c mice (data not shown), dysregulation of lymphocyte trafficking is likely a significant contributing factor to the development of TID in NOD.

The elevated CXCL12 expression may promote disease progression in NOD mice through its effect on Treg trafficking, which is partly regulated by CXCL12-CXCR4 interaction. A large body of evidence suggests that Tregs play a critical role in suppressing autoimmunity [36]. In NOD mice, the frequency and function of Foxp3^+ ^Tregs were reported to decrease with age [37]. We show that more Tregs were present in the bone marrow of NOD mice than age-matched BALB/C mice. Following AMD3100 treatment of NOD mice, the number of Tregs was significantly decreased in the bone marrow whereas the number was significantly increased in the spleen (see Additional file 4). However, because the number of Tregs in the bone marrow is only about one tenth of that in the spleen, Treg mobilization from the bone marrow alone cannot account for the significant increase in Treg numbers in the spleen. Regardless where the splenic Tregs come from, because a threshold ratio of Tregs to autoreactive T cells is important for Treg suppression of autoreactive T cells [6,38], sequestering Tregs in the bone marrow or some other organs in NOD mice may tip the balance in favor of autoreactive T cells.

The elevated CXCL12 expression also leads to retention of hematopoietic stem cells in the bone marrow of NOD mice. Because bone marrow-derived stem cells initiate pancreatic regeneration [39], it is possible that sequestering HSC and possibly other stem/progenitor cells in the bone marrow might contribute to disease progression in NOD mice by limiting their availability for islet regeneration. In support of this notion, systemic administration of CXCL12 ameliorates diabetes [40], perhaps partly by mobilizing HSC from the bone marrow to the periphery. The same mechanism may partly explain the effectiveness of CFA plus adoptive transfer of splenocytes, which contain a significant number of HSC and probably other progenitor cells [41], in curing diabetic NOD mice through regeneration of islets [42-45].

AMD3100 was originally developed to treat HIV infection through its antagonism of the CXCR4, a co-receptor for the virus [17]. Currently, it is being developed as an agent to mobilize HSC from bone marrow to peripheral blood for transplantation of stem cells in patients with non-Hodgkin's lymphoma or multiple myeloma [46]. We found that AMD3100 treatment of prediabetic NOD mice significantly delays insulitis and the onset of diabetes. In contrast, a recent study reported that AMD3100 treatment promotes diabetes development in a model where diabetes was induced by adoptive transfer of splenocytes from female NOD mice into sublethally irradiated male NOD mice [47]. In the transfer model, transferred T cells undergo homeostatic proliferation and acquire effector functions [30-32]. Irradiation of the recipient mice also introduces additional factors and processes which are not active in the non-irradiated prediabetic mice. Thus, the disease induction mechanisms in the transfer model could be significantly different from those in un-manipulated female NOD mice. Although the precise mechanism underlying the discrepancy between the two studies has yet to be determined, our finding provides a basis for further exploring the use of AMD3100 to prevent and/or treat TID and possibly other autoimmune diseases in patients with elevated CXCL12 or CXCR4 expression.

## Conclusion

Elevated CXCL12 expression in the bone marrow of NOD mice likely promotes development of TID by altering T cell and hematopoietic stem cell trafficking. The findings suggest the possibility of preventing and/or treating TID in humans by modulating either the expression or function of CXCL12 and CXCR4.

## Methods

### Mice

NOD mice from Taconic Farms were bred in our facilities. Female NOD mice were used in all studies and monitored for diabetes by checking the urine glucose level at least two times per week, starting around 12 week of age. Mice were considered diabetic when urinary glucose level reached 500 mg/dl, as measured with Diastix (Bayer Diagnostics). In our facility, approximately 95% of untreated NOD female mice became diabetic by 7-months of age. Balb/c mice were purchased from the Jackson Laboratory. EA16NOD mice were a gift from Drs. Diane Mathis and Christopher Benoist. *Cxcr4*^*f*/*f *^Lck-Cre mice and littermate *Cxcr4*^+/*f *^Lck-Cre were on the mixed 129/C57BL/6 background and were generated by breeding *Cxcr4*^+/*f *^Lck-Cre mice. All mice were kept under specific pathogen free facilities. For AMD3100 treatment, prediabetic NOD mice were given AMD3100 (5 mg/kg) subcutaneously daily for the indicated length of time. For analysis of T cell distribution, the mice were sacrificed after 2 hour of last AMD3100 injection. All animal studies are approved by the institutional Committees on Animal Care.

### Antibodies and flow cytometry

Single-cell suspensions were prepared from the spleen, lymph nodes and bone marrow. All staining antibodies were purchased from BDbioscience, except anti-Foxp3, lineage markers (Lin), and c-Kit and Sca-1, which were from E-bioscience, Stem Cell Technologies, and Biolegend, respectively. Cells were stained in the presence of 2.5 μg/ml anti-FcR antibody in PBS containing 0.1% BSA and 0.1% NaN_3 _and analyzed on a FACScaliber or a LSR II or a FACSAria, collecting 10,000 to 1,000,000 live cells per sample. Analyses were carried out with Flowjo software. For analysis of LSK cells, 10% of total cells with low level of lineage marker expression were gated for further analysis of Sca-1 versus c-kit expression profile. Intracellular staining of Foxp3 and Ki67 was performed according to the manufacturer's instruction.

### Immunohistochemistry

The pancreata were fixed in 10% formalin, embedded in paraffin, and sectioned. Paraffin-embedded tissue sections were stained with haematoxylin and eosin, and parallel sections were stained with polyclonal guinea pig anti-insulin (Zymed) or anti-glucagon antibodies (Linco Research Inc.), followed by incubation with ABC systems Kit (Vector laboratories). For destructive insulitis (loss of insulin staining), insulin-stained sections of pancreata were matched to serial sections stained for glucagon. Insulitis is defined as presence of infiltrating lymphocytes in islets regardless whether they stained positive for insulin or not.

### Chemokine gene expression

RNA was isolated from bone marrow using Trizol (Invitrogen), reverse transcribed using the Ampho-Labeling kit (Superarray, Frederick, MD), and the cDNA was labeled with biotin-16-dUTP (Roche). Cytokine expression was assessed using an Oligo GEArray^® ^Mouse Chemokines & Receptors Microarray (Superarray, Frederick, MD). Real-time PCR for CXCL12, CCL19, and GAPDH transcripts was performed with the probes and master mixture kit from Applied Biosystems.

### Homing of T cells

T cells were purified from lymph nodes and spleens from 12–16 week old NOD and Balb/c mice by negative depletion using a MACS and staining with biotinylated antibodies specific for CD11b, CD11c and B200 and subsequently with streptavidin-microbeads. The purified T cells (> 95% purity) were labeled with 1.5 μM CFSE, washed and then suspended in HBSS solution. 20 or 50 × 10^6 ^CFSE-labeled cells were transferred into the NOD and Balb/c mice. After 2 hr (for mice transferred with 50 × 10^6 ^cells) or 48 hr (for mice transferred with 20 × 10^6 ^cells) recipients were sacrificed, and the frequency of transferred CD4 T cells was determined in the bone marrow, lymph nodes and spleen by flow cytometry assaying for CFSE and CD4. To correct for differences in the input cell numbers among individual recipient mice, the homing index (HI) was calculated: HI = [% CFSE^+ ^CD4 T cells in BM or LN]/[% CFSE^+ ^CD4 T cells in spleen].

### Statistics

Statistical analysis for significance was done with either a two-tailed Student's t-test or a Kaplan-Meier product limit estimation. Error bars shown in all figures are one standard deviation.

## List of abbreviations

CXCL12: chemokine (C-X-C motif) ligand 12; SDF-1: stromal cell-derived factor-1; CXCR4: CXC chemokine receptor 4; CFA: complete Freund's adjuvant; G-CSF: granulocyte colony-stimulating factor; HSC: hematopoietic stem cells; TID: type I diabetes.

## Authors' contributions

QL conceived of the study, designed most of the experiments, collected data, analyzed and interpreted data, performed statistical analysis, and drafted the manuscript; YN collected and analyzed data on *Cxcr4*^*f*/*f *^Lck-Cre mice; YZ analyzed and interpreted data from *Cxcr4*^*f*/*f *^Lck-Cre mice; JC coordinated the study, participated in the experimental design, analyzed and interpreted data, and drafted the manuscript. All authors read and approved the final manuscript.

## Supplementary Material

Additional file 1 Figure s1 Click here for file

Additional file 2 Figure s2 Click here for file

Additional file 3 Figure s3 Click here for file

Additional file 4 Figure s4 Click here for file
