# Supplementary figures and images for: Elevated CXCL12 expression in the bone marrow of NOD mice is associated with altered T cell and stem cell trafficking and diabetes development
Source: BMC Immunol. 2008 Sep 15;9:51. doi: 10.1186/1471-2172-9-51 (PMC2556327; doi:10.1186/1471-2172-9-51)

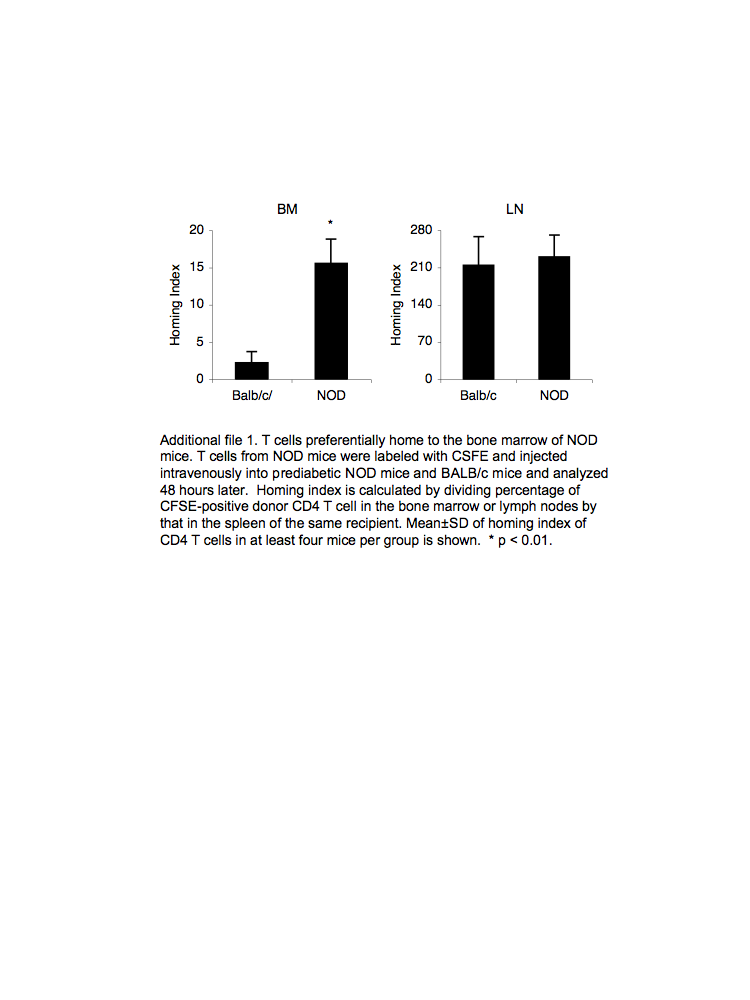

Supplement: Additional file 1 — Figure s1 [file 1471-2172-9-51-S1.tiff]

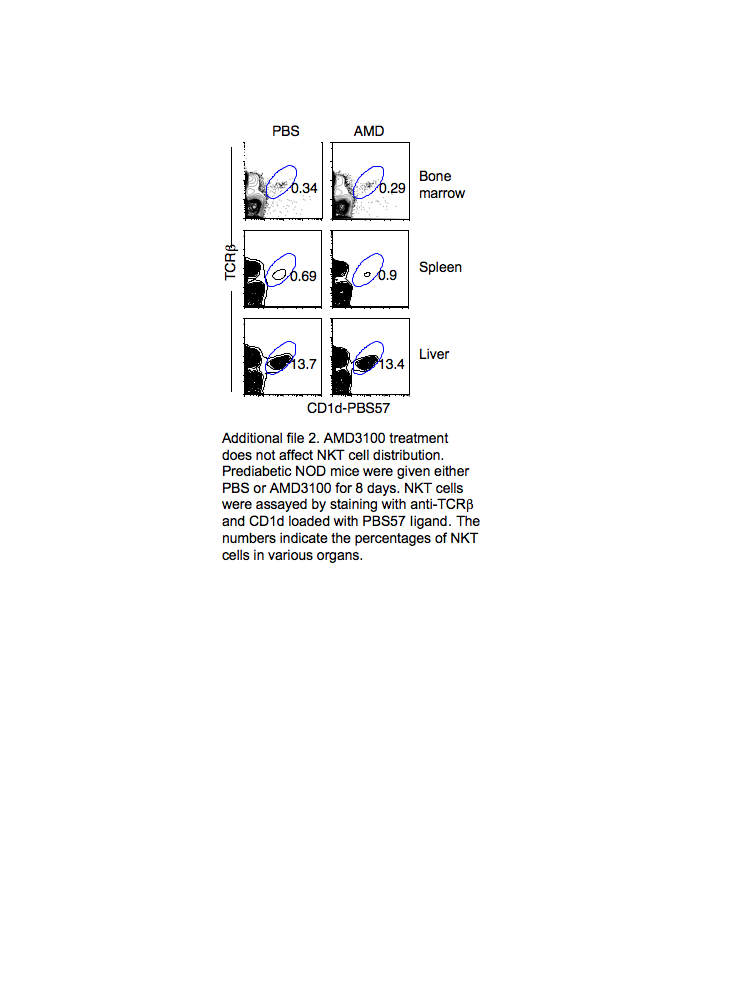

Supplement: Additional file 2 — Figure s2 [file 1471-2172-9-51-S2.tiff]

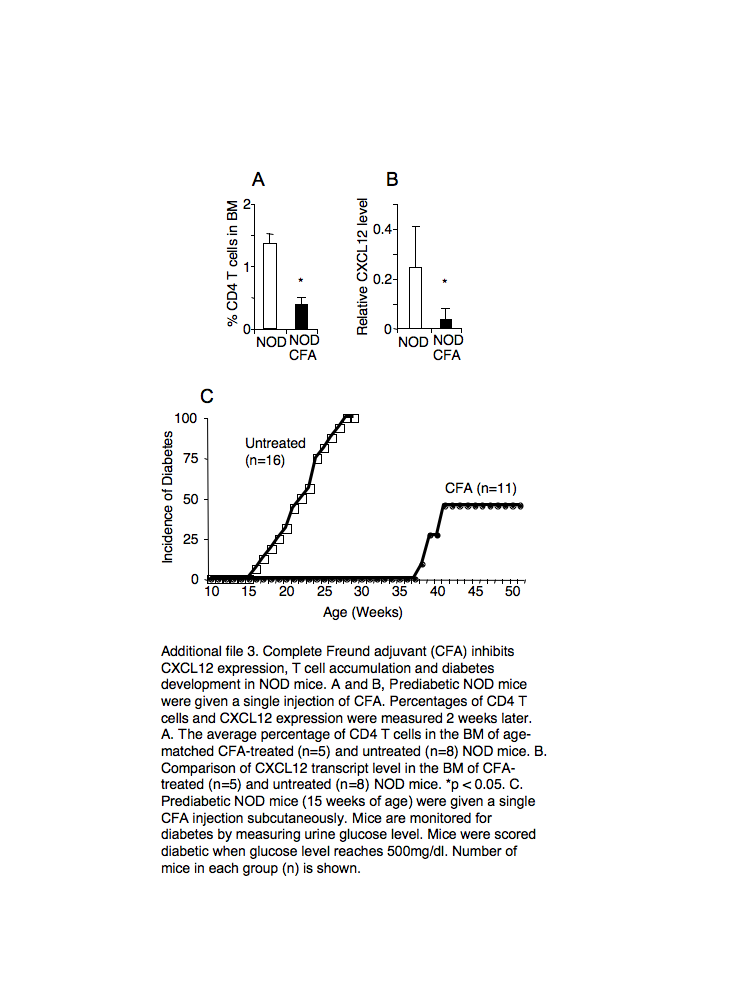

Supplement: Additional file 3 — Figure s3 [file 1471-2172-9-51-S3.tiff]

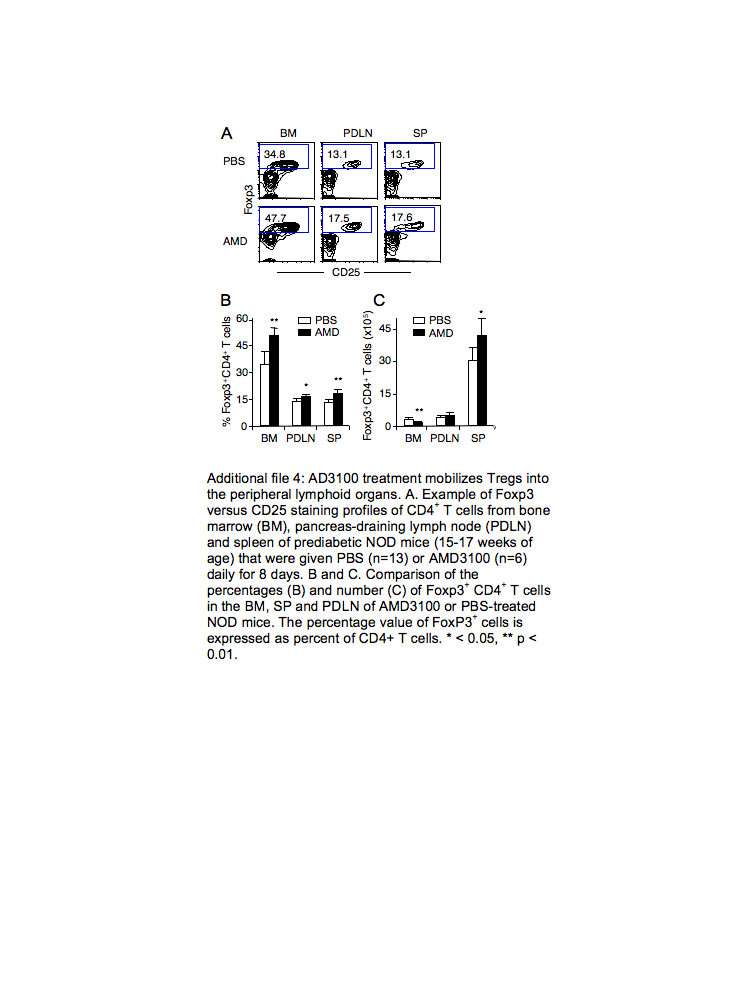

Supplement: Additional file 4 — Figure s4 [file 1471-2172-9-51-S4.tiff]
